# A qualitative study of risk and resilience in young adult women with a history of juvenile-onset fibromyalgia

**DOI:** 10.1186/s12969-021-00628-9

**Published:** 2021-08-17

**Authors:** Morgan Daffin, Mary K. Lynch-Milder, Robert C. Gibler, Caitlin Murray, Carly M. Green, Susmita Kashikar-Zuck

**Affiliations:** 1 Department of Pediatrics, Division of Child & Adolescent Psychiatry and Psychology, Norton Children’s Medical Group, University of Louisville School of Medicine, 200 East Chestnut Street, Louisville, KY 40202 USA; 2 Department of Psychiatry, Indiana University School of Medicine, Indianapolis, USA; 3 Department of Psychology, University of Cincinnati, Cincinnati, USA; 4 Division of Behavioral Medicine & Clinical Psychology, Cincinnati Children’s Hospital Medical Center, Cincinnati, USA; 5 Center for Child Health, Behavior & Development, Seattle Children’s Research Institute, Seattle, USA; 6 Department of Pediatrics, University of Cincinnati College of Medicine, Cincinnati, USA

**Keywords:** Juvenile-onset fibromyalgia, Fibromyalgia, Resilience factors, Risk factors, Young adults, Chronic pain

## Abstract

**Background:**

Juvenile-onset Fibromyalgia (JFM) is a chronic pain condition characterized by widespread musculoskeletal pain, fatigue, sleep difficulties, mood concerns, and other associated symptoms. Although diagnosed in childhood, JFM often persists into adulthood can result in continued physical, social, and psychological impairment. The purpose of this qualitative study was to identify themes of risk and resilience for long-term outcomes among young adults diagnosed with JFM in childhood.

**Methods:**

The sample included 13 young adults (ages 26–34) who had been diagnosed with JFM in adolescence. Focus groups were used to elicit qualitative information about living with JFM and perceived challenges and buffering factors impacting their adjustment.

**Results:**

The majority of participants (80%, *N* = 12) continued to meet criteria for fibromyalgia (FM). An iterative, thematic analysis revealed themes of resilience (e.g., greater acceptance, re-setting expectations, active coping, addressing mental health) and risk (e.g., lack of information, stigma, isolation, negative healthcare experiences).

**Conclusion:**

Results suggest the need for longer follow-up of youth with JFM as they transition to adulthood with multidisciplinary care and more attention to education about JFM and associated symptoms such as fatigue, as well as ongoing support for coping and mental health needs. A holistic approach to care during the transition years could be beneficial to minimize impact of JFM on long-term functioning.

## Background

Juvenile-onset Fibromyalgia (JFM) is a chronic pain condition characterized by widespread musculoskeletal pain, fatigue, mood concerns, and other associated symptoms [1–6]. JFM affects 2–6% of school-age children [7–11] and commonly persists into adulthood [12]. Consistent with studies highlighting the impact of pediatric chronic pain on long-term academic and vocational outcomes [13, 14], recent longitudinal data suggest that youth with JFM continue to experience impairment in physical, social, and psychological domains well into early adulthood [15].

Although pain severity tends to decrease over time among those with JFM, a trajectory of increased depressive symptoms appears to place a subset of individuals at greater risk for long-term functional impairment. A longitudinal study that followed over 100 youth diagnosed with JFM in adolescence identified three distinct trajectories: *Low-Stable* Depression, *Improving* Depression, and *Worsening* Depression based on symptom changes over an 8 year follow-up period [15]. The *Worsening* Depression trajectory showed worsening physical impairment into adulthood. In contrast, those in the *Low-Stable* and *Improving* groups did not experience worsening pain-related disability over time. These results clearly suggest that some individuals with JFM may be at-risk for worsening emotional and physical functioning, while others demonstrate greater resilience.

Research among adults with FM indicates that factors such as a controlling family environment [16], hypervigilance, and low perceived social support [17] increase risk for worse long-term functioning. In the general chronic pain literature, evidence suggests that peer support may buffer against the negative effects of pain on functional disability, emotional regulation capacities, and identity formation [18]. In contrast, optimism [19], having a sense of purpose [20, 21], actively coping with symptoms [22], and pain acceptance [22–25] are associated with less disease burden and improved functioning in adults with chronic pain [21, 25]. The complex and debilitating nature of JFM underscores the need to better understand the specific processes that predict poorer long-term outcomes among these individuals, those which promote resilience and improved functioning over time.

The purpose of this study was to explore young adults’ perspectives about risk and resilience years after their diagnosis of JFM in adolescence. We conducted focus groups to elicit qualitative information about the impact of the diagnosis of JFM and challenging or mitigating factors they experienced while living with JFM. Thematic analysis of qualitative data was used to identify cohesive themes under domains of risk and resilience.

## Methods

### Participants

Thirteen young adults (ages 26–34) from the cohort of participants who completed the longitudinal study of youth with JFM [15] participated in this qualitative study. Detailed characteristics and inclusion criteria of the original sample are described in other published articles [12, 15, 26]. Individuals were eligible to participate if they completed the final wave of the longitudinal study (between 2012 and 2015). Using their last known contact information (mail, email or phone), we were able to reach 19 of 97 eligible participants. Of these 19 individuals, 15 agreed to participate, 14 completed questionnaires, and 13 completed questionnaires and focus groups. The remaining were unable to be reached because their contact information changed, they moved, or did not respond to a request to participate.

### Procedure

Participants were first introduced to the study via phone or e-mail. After consent was obtained electronically, participants were sent a link via REDCap to complete online measures. Five focus groups were then completed with 1–4 participants per group through in-person interviews or remotely via HIPAA compliant video communication platform (Zoom). Eleven participants completed focus groups remotely, and two participants completed an in-person focus group. Five focus groups were sufficient to achieve thematic saturation [27]. Participants were compensated with a $20 gift card. The hospital Institutional Review Board determined that this was an IRB Exempt study.

### Survey measures

#### Background information

Participants provided demographic information (age, sex, race, and ethnicity), highest level of education, work status and current job, marital/partner relationship status, number of children, and current medications or other treatments (e.g., physical therapy, psychotherapy, etc.) for pain or other FM symptoms.

#### Assessment of current FM symptoms

Participants completed the *Widespread Pain Index and Symptom Severity Checklist*, identifying the number of locations in which they experience pain and the severity of associated symptoms such as fatigue and sleep difficulties in the past 3 months to assess whether they met classification criteria for adult FM [28]. A numeric rating scale from the *Brief Pain Inventory* [29] was administered to assess participants’ average pain intensity in the past week using a 10-point rating scale (0 = no pain; 10 = worst pain imaginable).

### Focus group

A trained psychology interviewer (MD) supervised by a licensed psychologist (SKZ) led each focus group, which ranged from 45 to 75 min in duration and were audio recorded for later transcription. The psychology interviewer followed a semi-structured interview script designed to elicit participants’ lived experience with JFM and related symptoms. Interview topics included the initial diagnosis, participants’ understanding of the diagnosis when they were adolescents and over time, current physical and mental health, how FM affected their life goals, coping strategies, and factors that helped or hindered their adjustment.

### Data analysis

Focus group recordings were transcribed verbatim by a research assistant (CG). Identifying information in transcripts was removed prior to research team review and coding. Interview transcripts were analyzed in NVivo (Version 12) using inductive thematic analysis [30]. The research team identified text segments containing meaningful information related to living with JFM, created categories of similar content, linked categories as appropriate, and refined categories by selecting quotes that captured core themes [30].

Following the approach to categorizing qualitative data developed by Braun & Clarke [31, 32], we conducted thematic analysis in iterative stages using the guiding quality principles of Yardley [33, 34]. Accuracy of transcripts was verified and transcripts were read with detailed attention by all study team members. During the coding phase, MD and RG re-reviewed all transcripts, tallied the number of times specific content was expressed by participants, and began identifying codes. All potential codes were reviewed by the entire team to ensure reliability, and following initial coding of one transcript, were formed into a codebook within NVivo.

The coding process was comprehensive and iterative to ensure the extracted quotes were fully understood and grouped appropriately into themes. Themes were then analyzed inductively by the entire team to ensure the meaning of the participant’s lived experience was displayed in the overarching themes. Quotations were used to demonstrate how themes were grounded in the data.

## Results

### Demographic and clinical characteristics

Sample characteristics are summarized in Tables 1 and 2. Participants completing all aspects of the study were 13 young women aged 26–34 (11 White, 2 Black). Half the participants were married and living with spouses, and six had children. Most (71.5%) participants had earned a bachelor’s degree or higher, and 64.3% of the sample was working full-time.

**Table 1:** Demographic characteristics

Variable	Mean (SD) or N (%)
Age	29.9 (1.5) Range: 26.7–33.9
Sex (% female)	14 (100.0%)
Race	
White	12 (85.7%)
Black	2 (14.3%)
Ethnicity	
Hispanic/Latinx	1 (7.1%)
Non-Hispanic/Latinx	13 (92.9%)
Marital status	
Single	7 (50.0%)
Married	7 (50.0%)
Living situation	
With parents	3 (21.4%)
With roommates	1(7.1%)
With spouse	7 (50.0%)
With a boyfriend or girlfriend	1 (7.1%)
Alone	2 (14.3%)
Highest level of education	
HS diploma or GED	1 (7.1%)
Vocational/ trade/ associate’s degree	1 (7.1%)
Some college	2 (14.3%)
BA or 4-year college degree	7 (50.0%)
MA or professional degree	3 (21.4%)
Work	
Full-time	9 (64.3%)
Part-time	3 (21.4%)
Not currently employed	2 (14.3%)
Annual income	
< or = $30,000	5 (35.7%)
$30,001 – $50,000	4 (28.6%)
$50,001 – $75,000	2 (14.3%)
> $75,000	1 (7.1%)
Declined to answer	2 (14.3%)
Children (% yes)	6 (42.9%)

**Table 2:** Clinical characteristics

Variable	Mean (SD) or N (%)
Met criteria for fibromyalgia^a^	12 (80%)
Widespread pain index (WPI)	11.3 (4.0)
Symptom severity scale (SSS)	7.9 (2.5)
Average pain intensity (NRS 0–10)	3.4 (1.4)
Current healthcare services (% yes)	8 (57.1%)
Physical therapy	3 (21.4%)
Psychotherapy	4 (28.6%)
Other (massage, chiropractic)	5 (35.7%)
Current medication for pain/ FM sx (% yes)	4 (28.6%)
Current medication for mood sx (% yes)	7 (50%)

^a^Based on the published ACR guidelines, a participant was considered to meet classification for adult FM if they had: (1) WPI score ≥ 7 and SS score ≥ 5, or WPI = 3 to 6 and SS ≥ 9; (2) symptom duration of at least 3 months; and (3) no underlying medical condition that would otherwise explain the pain

Twelve participants (80%) currently met the 2010 American College of Rheumatology criteria for a FM classification [28]. On average, they reported low-to-moderate levels of pain (3.4/10) in the last week. Over half of participants (57%) were utilizing healthcare services at the time of study completion, including physical therapy (*n* = 3), psychotherapy (*n* = 4), and other services such as massage therapy or chiropractic services (*n* = 5). Four participants were taking medication for pain management, and 7 were taking medication for mood symptoms.

### Thematic analysis

Our iterative coding process yielded 53 initial codes, each capturing participants’ distinct experiences. These were distilled into a smaller collection of codes which best reflected themes representing *resilience* and *risk*. *Resilience* themes captured aspects of participants’ personal attributes or life experiences that enhanced their ability to cope with JFM, whereas *risk* themes encompassed factors which interfered with participants’ ability to cope with JFM. The final codes, risk and resilience themes, and exemplar quotes from participants are displayed in Tables 3 and 4.

**Table 3:** Resilience

Theme	Code	Example Quote
Learning to live with fibromyalgia by embracing the growth mindset	Altering expectations	“Now that I am in a position where I am working from home, I can set my own schedules, set my own pace … I definitely feel like it’s been so much more manageable.”
	Actively seeking out answers	“It definitely was a trial and error period of seeing what actually did help outside of medication.”
	Self-advocacy	“I’m a very independent person by nature. But, obviously, there are situations when I absolutely cannot do something or just need help. And so I feel like I have gotten a lot better about asking for help when I need it.”
	Empathy for others	“It really gives you pure empathy for other people who are suffering.”
	Benefit finding	“It’s also made me appreciate small things.”
	Self-compassion	“Patience for myself and for other people around me, or like not being too hard on myself.”
Managing FM holistically	Finding effective non-pharmacological interventions	“Just emphasizing that it is multi-faceted. You can’t just tackle one side of it, you have to come around and take a holistic approach.”
	Education on the mind-body connection	“It was that explanation [of the pain gate, pain-stress cycles,] that kind of helped things click for me.”
	Helpfulness of mental health treatment	“The therapist really helped me realize that it doesn’t matter if I resent my body. It’s still my body, and I’m going to have to make it work.”
	Exercising to manage symptoms	“Now I’m better at getting that sweet spot of moderate exercise that helps with all the stiffness, and stuff that doesn’t make me flare up because I overdid it.
Perseverance	JFM is not the end	“There was no diagnosis that was going to stop me.”
	Internal resolve to prove capability	“I’m an adult, so I can deal with it.”
Social support and validation	Relief with diagnosis	“I would have constantly wondered what’s wrong with me.”
	Validation from others	“You kind of want that explanation or validation like, ‘No you’re not crazy. There is something wrong.’”
	Support from others	“Surround yourself with people who are going to keep pushing you, because there are days that you can get really down and out. You need those people to pull you out of it.”

**Table 4:** Risk

Theme	Code	Example Quote
Unequipped to deal with long-term, complex nature of FM	Delayed acceptance of diagnosis	“It wasn’t until probably I was about 28, did I really start to be like, ok, this is obviously going to be with me for the rest of my life.”
	Difficulty of teenage years	“As a teen, I thought my life was over.”
	Dealing with depression and anxiety	“I have pretty bad anxiety. I struggled with depression throughout college.”
	Fatigue symptoms need more attention	“The pain I can kind of mitigate, though I’m not the best at it, but the fatigue overrides everything.”
	Long-term impact on life	“It is multifaceted. We do need to encourage our patients to – you know, they will have to change their entire lives.”
	I wish I knew more when I was younger	“It wasn’t until college that anyone sat down and talked to me about pain management strategies.”
Inadequate support and stigma	Negative stigma associated with diagnosis	“I had to hide it.”
	Burden on others	“Because I couldn’t do anything, I was dependent on everyone. There was so much pain, and I wasn’t able to keep myself distracted … I couldn’t even care for my son.”
	Social isolation	“I got to a point where I got too exhausted to really have friends.
	Poor parenting (poor mental health, overprotective parenting)	“My parents want to kind of put me in a bubble at this point in my life and not let me do anything. ‘You’re gonna hurt yourself.’”
Negative healthcare experiences	Dismissive providers	“I was tired of getting the look, like, ‘Oh, you sure?’”
	Medication was not the only answer	“For basically all of high school, it was, ‘You have this disease, you’re going to be in pain pretty much every day for the rest of your life. … here’s some Cymbalta ..’ And that was not super helpful. Especially to a teenager who has so much else going on, too.”
	Poor access to quality care	“My doctor didn’t really understand how to manage my condition, because he didn’t really understand it either.”

#### Resilience themes

##### Learning to live with FM by embracing a growth mindset

Participants shared that learning to effectively cope with JFM required gradual adjustments to their daily lives and goals which unfolded over the course of many years.

This process was “*a trial and error period of seeing what did help outside of medication*.” For many participants, one key to managing their symptoms was learning to advocate for their needs in personal relationships and with medical professionals, and seeking a more supportive provider/health care team if they did not feel comfortable with their current provider/s.

Participants reported that, with time, living with FM helped them practice self-compassion and shift their thought patterns and beliefs to move toward greater acceptance of their circumstances as they matured. *“*[I’ve found] *patience for myself and for other people around me*.” Many participants also described an enhanced ability to extend empathy toward others. *“I think I’m more sympathetic*. *.. and have a little more patience*.”

Other participants described finding benefits in “small” things, including becoming more attuned to their bodies. *“It took a period of 6 months to a year of me just exercising regularly to understand: OK, have I really caused a flare-up, or am I just uncomfortable?* This process of adjustment took years with many small incremental changes over time.

##### Managing JFM holistically

Managing JFM required exploring an array of treatments. One participant stressed that “*just prescribing pain medication wasn’t the answer*.” There was agreement among participants that managing pain required a holistic approach that considered relationships among pain, mood symptoms, and lifestyle. One participant appreciated a health care visit where they received such an explanation, stating that “*The thing I remember most from that meeting is they did describe the cycle* [of pain]*, and they drew on the white board: mental health, depression, sleeplessness, appetite, and pain. And they’re like, ‘they all correlate to each other, so if one is off balance, the rest gets affected.’”*

While participants’ ability to achieve “balance” to manage JFM was highly individualized, participants agreed that addressing both mental and physical health was critical. Participants who received mental health treatment as teenagers indicated that they benefited greatly from hearing healthcare professionals describe the mind-body connection. *“The therapist really helped me realize that it doesn’t matter if I resent my body. It’s still my body, and I’m still going to have to make it work.”* Other participants found engaging in frequent physical activity to be beneficial. “*It wasn’t until I went to college and started running and exercising and managing my pain in a different way that I actually felt like a real person again.”*

##### Perseverance

Many participants expressed resolve to continue living their lives in the fullest despite the limitations of JFM. This seemed motivated by internal convictions and a refusal to give up in the face of hardship. Multiple participants used similar expressions to communicate the possibility of living a meaningful, productive life despite pain. *“*[JFM] *is not a death sentence. There’s going to be work to put in, but don’t fight it … Seek support and help. It’s going to be overwhelming at first, but once you get a handle on it and see what works for you, just keep doing it and stick with it … You can get out of life what you want to put into it. Just knowing that you’re going to have to try a little harder than everybody else does. Don’t let that define you*.” When asked for advice they would give to others living with JFM, many participants again expressed internal resolve. “*Let them know that they can still lead a normal* [life]*, or the life they would want to live*.”

##### Social support and validation

Participants reported an extended time spent looking for an explanation for their symptoms, but without a “name” or clear diagnosis. Many sought additional opinions from medical professionals and underwent an array of medical tests and procedures before receiving a JFM diagnosis. Receiving confirmation that their symptoms were recognized in medical nomenclature provided a sense of relief for many participants. “*I was more excited or elated* [to be diagnosed]*, because I knew there was something. It wasn’t just me*.” Much of this relief stemmed from the validation that others believed their symptoms were real. *“It was a little bit of a relief to be – like somebody actually said, ‘I’m acknowledging that you’re in pain a lot.’ And that it wasn’t something I’ve made up to get attention.”*

Participants also reported gratitude for the validation received from loved ones and their emotional and instrumental support. “*My husband wanted to be an exercise physiologist, so he started writing me programs that would be manageable for me.”* Other participants emphasized the importance of familial social support. “*I always had family I could fall back on*.”

#### Risk themes

##### Unequipped to deal with the long-term, complex nature of FM

Participants uniformly stated that adjusting to their FM diagnosis was a taxing process that unfolded over many years. For some, acceptance of their diagnosis was a process in and of itself. *“I think for me, for my goals and aspirations,* [FM] *was a diagnosis that didn’t fit into what I saw myself doing in the future.”* Once they acknowledged their diagnosis, it seemed like it was easier for them to begin the process of adjustment.

Many participants disclosed that they experienced depression, social isolation, and difficulties with peer relationships as teenagers. Some endured these challenges without seeking treatment, which may have prolonged their suffering. *“I was severely depressed. I felt very alone. I didn’t know how to cope.” “In high school,* [FM] *kind of made me not want to go to school.” “I felt really lonely as a teenager, since I was the only one I knew who had this.”* For some, the pressures of their educational and vocational pursuits may have interfered with attending to managing their FM symptoms. “[In college]*, I was more in a phase of grin and bear it. Plus, dealing with the mental stuff … I had a lot of things to distract me from* [accepting the FM diagnosis].”

Some participants did not recall learning about how nonpharmacological strategies could help them manage pain, fatigue, and mood symptoms. “*It wasn’t until college that anyone sat down and talked to me about* [non-drug] *pain management strategies*.” There was consensus among participants that fatigue, anxiety, and depressive symptoms were underemphasized by providers, and they felt they had very few tools at their disposal to manage these aspects of JFM. *“If you develop FM, you will get the depression, and the anxiety.* [I would tell providers to] *let them [patients] know and maybe guide them to help them a little more. Because I just got the, ‘Oh, here’s your fibromyalgia.’ And that was it.”*

Participants reported that they did not fully understand that their symptoms would persist and could impact their lives once they became adults. “*It wasn’t until I was about 28 that I started to be like, ‘Ok. This is obviously going to be with me for the rest of my life.*’” Some participants suggested that the burden of their symptoms may have lessened had they been equipped with knowledge about the multi-faceted nature of FM in childhood. “*I think because I neglected my FM diagnosis for so long,* [seeking proper treatment] *took me years … Like, not until this year did I seek mental health help. If I had acknowledged my diagnosis earlier, I might have sought that help a lot earlier*.”

##### Inadequate support and stigma

Many participants shared about a perception that important people in their lives – including peers and medical providers – viewed their diagnosis as fraudulent or attention-seeking in nature. *“You look young and normal. Nothing is wrong.”* “*… You miss a lot of school and whatnot, and people start looking at you like you’re kind of crazy.”* Many participants also emphasized the impact of stigma on their vocational prospects and employers’ perceptions of them. “[Employers] *just think you’re lazy. They don’t listen to your explanation.”* “[It is] *incredibly frustrating, and almost demoralizing, to have to justify my pain to* [my boss].”

Symptoms of FM also interfered with participants’ personal relationships. Many participants felt, and continued to feel, isolated from others due to their symptoms. *“I got to a point where I got too exhausted to really have friends.”* Unfortunately, even when they were with friends or family members as young adults, participants reported feeling like they were a burden and felt a great deal of guilt. “*I was so dependent on everyone. There was so much pain. I wasn’t able to keep myself distracted because I couldn’t do anything, to the point where I couldn’t even care for my son*.”

##### Negative healthcare experiences

Many participants perceived that their healthcare was negatively impacted by some providers’ dismissive attitudes towards their symptoms and questioning their motivations for seeking treatment. “*You’re just using this as a catch-all to not deal with the individual issues you’re having. We don’t believe* [FM] *is a real diagnosis*.” Some also believed that many providers lacked specialized knowledge about treating FM. “*My doctor didn’t really understand how to manage my condition, because he really didn’t understand it either.*” Participants often heard the message that they just needed to take medication or “exercise more” to manage symptoms, though these treatments failed to sufficiently address the mental health and fatigue aspects associated with FM. *“For basically all of high school, it was, ‘You have this disease. You’re going to be in pain pretty much every day for the rest of your life. Here’s some Cymbalta. And that was not super helpful, especially to a teenager who has so much else going on, too*.” Finding healthcare professionals who were knowledgeable about treating JFM was often difficult but highly valued when participants were able to connect with the right health care team.

## Discussion

This qualitative study explored themes of risk and resilience among 13 young adults diagnosed with JFM in their youth by eliciting qualitative information about their lived experience of JFM over the years as they transitioned into young adulthood. On average, participants had suffered from JFM symptoms for over a decade, and the majority met adult criteria for FM at the time of this study. Over half of participants continued to seek healthcare services for FM symptoms, and a substantial proportion were using medications for pain management and mood concerns.

Thematic analysis revealed that individuals with adolescent-onset FM experienced adjustment to living with the condition as an ongoing, long-term process. Many participants described initial difficulty with accepting the diagnosis and understanding the scope of the multi-faceted impact of the condition. This delayed process of acceptance may be related to the difficulty of receiving this diagnosis at a vulnerable developmental age (i.e., during adolescence) along with the medical uncertainty surrounding the diagnosis itself.

Nearly all participants reported feeling unequipped to manage pain alongside commonly co-occurring symptoms such as fatigue, anxiety, and depression. These symptoms may represent key risk factors for negative prognostic outcomes for overall health status, including pain and functional disability [28, 35–37]. In contrast, participants who learned to cope effectively reported a refusal to allow FM symptoms to hold them back from achieving goals, an embracing of optimism, and a rejection of their JFM diagnosis as a core feature of their identity. This resilience factor captures the extent to which an individual believes their life has direction [20, 21]. Benefit-finding and gaining positive perspectives appears to facilitate healthy coping with chronic conditions [38].

The social impact of JFM in adolescence was substantial among our participants. Several individuals reported challenges in their close relationships following the onset of pain [39, 40]. Adults with chronic pain frequently experience difficulties with creating lasting friendships [41], and diminished social support intensifies associated psychological distress [42, 43]. Many participants also encountered providers who seemed dismissive, which is similar to a common theme of stigma expressed by adults with chronic pain [44–46]. In contrast, participants shared that coping with FM was made easier when they felt supported and validated by family and healthcare providers. This is consistent with research indicating an association between social support, mood difficulties and pain intensity in adults [47].

### Treatment and research implications

This qualitative investigation deepens our understanding of the experiences of youth with JFM who are transitioning into young adulthood. From a clinical care standpoint, participants emphasized the need to expand psychoeducation about FM, implement coping skills early in order to mitigate the long-term consequences of associated symptoms, and receive clear messages from providers about correct terminology and diagnostic labels. Unfortunately, the healthcare system is currently not ideally equipped to deal with transitioning adolescents with chronic pain from pediatric care to adult health care, and these gaps need to be addressed. Our findings suggest that multidisciplinary approaches that incorporate non-pharmacological treatment options and emphasize mental health care may beoptimal for youth with JFM. This is consistent with research indicating that as young people transition from pediatric to adult care, collaboration between stakeholders results in positive outcomes and reduced anxiety [41, 48].

Patients with JFM may benefit from early interventions that promote a mastery of coping skills, enhance self-efficacy, and encourage developmentally-appropriate levels of functioning despite pain. Although skills-focused cognitive-behavioral interventions are effective in reducing pain and depressive symptoms in JFM [49, 50], elements of Acceptance and Commitment Therapy – which focus on increasing values-driven action and decreasing “fusion” with distorted conceptualizations of the self [51] – may “bridge the gap” between short-term symptom reduction and long-term functional outcomes. Medical providers can help facilitate resilience and coping by validating the patient’s experience, providing clear education about FM and associated symptoms, establishing realistic expectations for symptom management over time, encouraging functioning despite pain, focusing on motivation to return to developmentally appropriate activities, pointing out benefits of social engagement, and encouraging use of evidence-based apps for pain coping (e.g. WebMAP mobile [52]). Further, because participants described the importance of becoming more attuned with their bodies, individuals with JFM may benefit from exercise-based interventions that improve body awareness, reduce fears of pain and injury, and promote exercise that is safe to engage in regularly. Group interventions that improve patients’ social connectedness, perceived validation, and skill in communicating their needs may also be valuable additions to pain coping skills training.

## Conclusions

This study is the first to our knowledge to employ rigorous thematic analysis to explore the life experiences of young adults diagnosed with JFM in adolescence. Future cross-sectional and longitudinal research is needed to examine the risk and resilience processes described by participants and improve the field’s understanding of the factors most strongly associated with or predictive of functional outcomes for youth with JFM. However, despite the strengths of this study, several limitations should also be considered. The current sample was a small subset of participants from the longitudinal study by Kashikar-Zuck and colleagues [15]. As almost all met criteria for adult FM, it is possible that this group had more severe symptoms than the larger sample. Additionally, our sample included predominantly Caucasian females. Although this is representative of treatment-seeking pediatric patients with FM, additional studies with larger and more diverse samples will ensure the generalizability of our findings.

In conclusion, results of this study advance our understanding of the perspectives of young adults with FM and the factors that may contribute to their outcomes in early adulthood. Findings provide valuable insights into risk and resilience factors that could promote well-being as youth with JFM learn to manage their symptoms and cope with the developmental challenges of the transition to adulthood.

## Data Availability

The data that support the findings of this study are available in de-identified form from senior author, Susmita Kashikar-Zuck at Cincinnati Children’s Hospital (susmita.kashikar-zuck@cchmc.org) by qualified individuals upon request for scientific purposes under a formal data-sharing agreement signed by the relevant institutions/individuals.

## Abbreviations

JFM: Juvenile-onset fibromyalgia
FM: Fibromyalgia
